# How Do Urban Land Expansion, Land Finance, and Economic Growth Interact?

**DOI:** 10.3390/ijerph19095039

**Published:** 2022-04-21

**Authors:** Ke Zhao, Danling Chen, Xupeng Zhang, Xiaojie Zhang

**Affiliations:** 1 Department of Land Resourse Management, College of Public Administration, Huazhong Agricultural University, Wuhan 430070, China; ccnuzhaoke@163.com (K.Z.); danlingchen@mail.hzau.edu.cn (D.C.); 2 Department of Land Resource Management, College of Public Administration, Huazhong University of Science and Technology, Wuhan 430074, China; xupengzhang@hust.edu.cn; 3 Department of Public Administration, School of Humanities and Law, Northeastern University, Shenyang 110169, China

**Keywords:** land economics, urban land expansion, land finance, panel-data vector autoregressive (PVAR) model

## Abstract

Land finance has consumed a lot of China’s urban land resources while contributing to its economic growth. Urban land expansion, land finance, and economic growth have attracted significant scholarly and social attention. However, the influence mechanisms among them have not yet been fully investigated. Based on a conceptual framework analysis, in this study, the panel unit-root test, system-GMM, panel Granger causality test, impulse-response analysis, and variance decomposition were used to analyze the interactional relationships among urban land expansion, land finance, and economic growth for 30 provinces in mainland China during the period of 2000–2017. The findings show that these three factors interact with each other. Land finance exhibits a positive effect on urban land expansion and economic growth. This result is further supported by the Granger causality tests. Moreover, the VAR Granger causality-test results show a unidirectional causality flowing from urban land expansion to economic growth. The impulse-response analysis also reveals that the responses of urban land expansion to shocks in land finance appear to be positive throughout the 10 periods, which is similar to the reaction of economic growth to shocks in land finance. The result of variance decomposition indicates that the explanatory power of urban land expansion for land finance increased from 0.20% to 1.90%. In contrast, the changes in economic growth made the lowest contributions to urban land expansion and land finance. The latter made the highest contribution to economic growth, with average contribution rate of 65.26%. The findings of this study provide valuable policy implications for China, heading for a high-quality development stage.

## 1. Introduction

From 1981 to 2018, the area of China’s urban construction land increased from 6720 to 56,075.9 km^2^, which is an astonishing annual growth rate of 5.90%. Moreover, the urban population increased from 201.71 million to 831.37 million, with an average annual growth rate of 3.90%. Land urbanization proceeded faster than population urbanization. Although urban areas comprise a very small percentage of the earth’s land surface area, their rapid expansion has created many problems, such as the loss of farmland and open spaces [1,2,3,4]; the depletion of scarce farmland resources, which threatens food security [5,6]; the loss and destruction of wildlife habitats [4,7]; a decline in and increased threats to biodiversity [7,8]; and more fragile eco-environmental quality and socioeconomic sustainability [9].

Land finance, operated by the local authorities, refers to the revenue, including land-transfer revenue, land taxes, and loans obtained by local governments using urban land as collateral [10]. It also refers to a fiscal phenomenon, which is characterized by a heavy reliance of local governments on land transfer revenue [11,12,13]. Land finance primarily involves the generation of revenue by buying and selling land assets at lower and higher prices. Land assets have become an important source of capital investments by subnational governments in developing countries [14]. Such financing can attract business investment and stimulate economic development, ultimately helping local government officials to be promoted to favorable positions. Land revenues account for 60–80% of local government revenues [12]; thus, land-use decisions are driven primarily by fiscal factors that encourage the expansion of urban built-up areas. To obtain additional land-transfer revenues, local governments supply more construction land in the market, thus depriving cities’ right to future development and creating more intergenerational inequality. Land finance has jeopardized China’s sustainable development by encroachment on farmland, the rise of real-estate market bubbles, and the creation of substantial risks to financial and economic stability [6,10,15].

Urban land expansion and land finance are two phenomena closely related to land conversion. In China, the special designs of the dual land ownership, land expropriation and compensation system, and land-transfer system have allowed local governments to exercise their rights to allocate land resources in order to obtain as much land revenue as possible and increase extrabudgetary incomes, thereby increasing investment capacity, which encourages economic growth but fuels urban land expansion. A comprehensive understanding of the relationships among urban land expansion, land finance, and economic growth has important theoretical significance to the formulation and optimization of land supply and transfer policies that control the excessive and rapid expansion of urban land.

The literature on the relationships among urban land expansion, land finance, and economic growth has focused on the following aspects:

(1) The relationship between urban land expansion and land finance: Some scholars have studied these factors by using the panel Granger causality-test method and found that land development is the Granger causality of the growth in local fiscal revenues [16]; in contrast, only a reverse causal relationship exists in the stage of advanced industrialization [17]. Land-transfer fees significantly encourage urban land expansion, whereas land taxes could theoretically hinder it [18]. Studies on the effects of land finance on urban land expansion have shown that the pursuit of land finance by local governments has encouraged the expansion of urban construction land [19,20,21].
(2) The relationship between urban land expansion and economic growth: The research in this field falls into two categories. The first one focuses on the contribution of land to economic growth. According to the literature, the urban construction land [22], the conversion of rural land to urban use [5], as well as the expansion of construction land [23,24,25], exhibit significant effects on economic growth. Yang et al., (2020) also found a positive relationship between the industrial land-transfer price and economic growth rate [26]. The second category considers economic growth as a driving factor of urban land expansion. Annual growth in GDP per capita drives half of the observed urban land expansion in China [7]. Tan et al. [27] and Liu et al. [28] obtained empirical results and demonstrated that the speed of urban land expansion is closely related to rapid economic development. However, Li et al. found a negative contribution of economic growth to the growth of construction land [29]. Urban spatial expansion arises mainly from the following three powerful forces: growing population, rising incomes, and falling commuting costs [1]. Urban sprawl has been significantly associated with urban population density, GDP per capita, and industrial structure [9].
(3) The relationship between economic growth and land finance: Since the 1990s, land finance has become increasingly important and continues to accelerate China’s urbanization and economic growth [13]. However, sustainable economic growth demands significant investments in infrastructure [30], which are provided as land-based finance to local governments [31,32] through the main channel of land conveyance revenue, which serves as a signal of the credit quality of the local governments [33], who have access to more loans from banks and investments for their economic advancement. Moreover, their reliance on land finance has bolstered the real-estate industry in China, making it a pillar industry [34]. However, Hou et al., (2021) showed that land finance has a significant inhibitory effect on the growth of the green economy [35]. Lin and Zhang (2015) found an inverse U-shaped relationship between the importance of land commodification to municipal finance and the level of urban economic growth [36].

Although it is well-known that the economic factors affecting the economy include consumption, investment, and export, the main driving force of economy in China is investment, with government investment accounting for a rather large proportion. Urban construction land is owned and supplied by the state. The local governments acquire the larger share of land finance, and tend to invest in infrastructure, thereby affecting economic growth. Therefore, this study focuses on the relationships among urban land expansion, land finance, and economic growth. However, most studies have failed to incorporate all three variables into one system. The lack of variables hinders a comprehensive understanding of the relationships among them and causes biases in econometric estimations. Studies on these relationships can address the following issues: (1) Whether urban land expansion in China is a result of the local governments’ pursuit of land finance or growth in demand due to economic development, or if it is derived from institutional and market forces; (2) In bolstering the real-estate industry, whether land finance results from urban land expansion, economic growth, or both; (3) Whether or not the expansion of urban construction land allocated by local governments and the resultant land revenues promote economic development.

**Figure 1:**
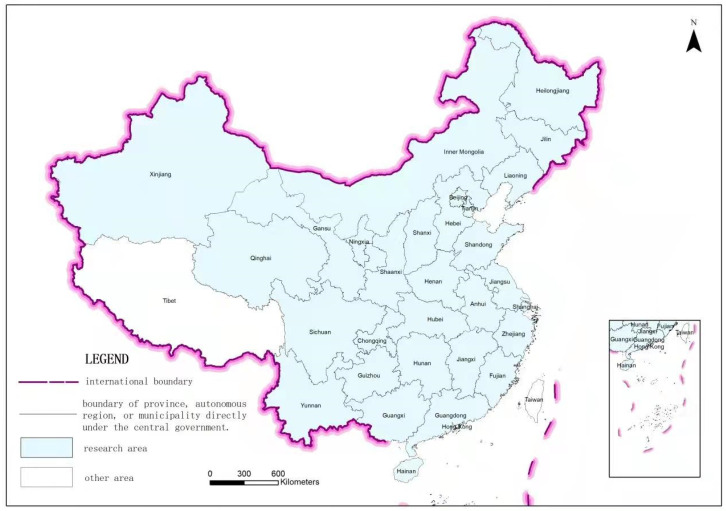
The spatial location of study area.

Using panel data from 2000 to 2017 for 30 provinces in mainland China (Figure 1), herein, a panel-data vector autoregressive (PVAR) model was employed to analyze the relationships among urban land expansion, land finance, and economic growth. This study offers a two-fold contribution to existing literature. First, the study provides a conceptual framework on the multiple causal relationship among urban land expansion, land finance, and economic growth, which has rarely been discussed in the literature. Second, the PVAR estimation was employed because of its capability to identify the dynamic effect of heterogeneity and the causal effects among urban land expansion, land finance, and economic growth. However, the interaction effects have been largely ignored in previous studies.

The remaining part of this paper is organized as follows: In Section 2, a systematic, theoretical, and analytical framework incorporating urban land expansion, land finance, and economic growth is proposed. Section 3 explains the methodology and data. Section 4 discusses the empirical evidence. Section 5 presents conclusions and policy implications.

## 2. Conceptual Framework

Urban sprawl and urban land expansion are similar concepts manifested as the continuously increasing size of construction land and continuously growing peripheral spaces. Urban sprawl is usually characterized by low density, single-land-use, dispersion, and leapfrog development [37]. However, urban land expansion refers to the increase in urban built-up land in the process of urbanization, which is not necessarily accompanied by low land efficiency. European and American scholars have paid more attention to urban sprawl; in contrast, Chinese scholars have traditionally been more interested in urban expansion; however, they have recently been focusing more on the effects of urban sprawl.

**Figure 2:**
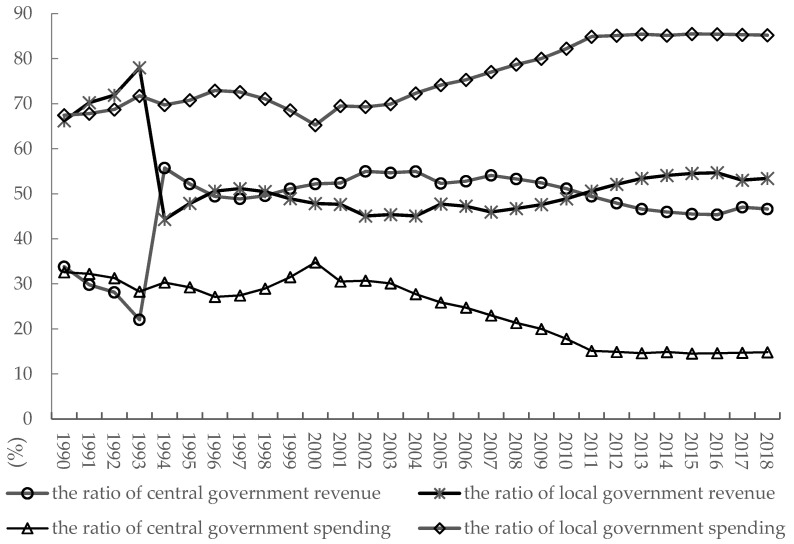
Proportions of revenues and expenditures of Chinese central and local governments from 1990 to 2018.

Land finance originated from the gap between local fiscal revenues and expenditures caused by the tax-sharing reforms of 1993. In recent years, the proportions of local governments’ fiscal revenues have been slightly higher than those of the central government, but local fiscal expenditures have been increasing and significantly exceeding the spending by the central government (Figure 2), resulting in higher local fiscal deficits. Under pressure from the GDP Competition Championship and fiscal deficits, local governments have actively been seeking new sources of finance, such as land fiscal revenues, within the legal tax system. Local governments have become over reliant on these sources [38,39,40]. A clear distinction should be made between land fiscal revenue and land finance. Land fiscal revenue already exists when there is land tax. Land finance refers to land-related income, which includes land-transfer revenues, land-related taxes, and land mortgages. Measurement indicators of land finance are usually divided into the following two categories: one is the total income derived from land-transfer revenues, land taxes, and land mortgages [6,41]; and the other is determined by relative scales.

**Figure 3:**
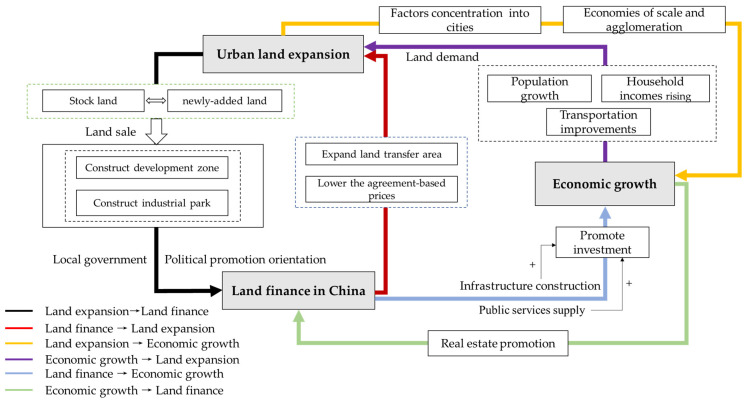
Mechanism of relationships among urban land expansion, land finance, and economic growth.

In this study, a conceptual framework was constructed to study the relationships among urban land expansion, land finance, and economic growth (Figure 3).

(1) To raise land finance, local governments can supply more plots of stock construction and newly expanded land to the market. Stock construction land includes land recovered (retracted) due to the expiry of rights, land requisitioned by the government, and land abandoned voluntarily by enterprises or individuals. The newly expanded land primarily consists of land expropriated from rural collectives. In general, state-run stock urban land plots are relatively close to commercial centers, whose surrounding superior infrastructures and locations offer higher prices than the plots of newly expanded land. Since the reform of the tax-sharing system, local governments have played crucial roles in accumulating land revenues and financing urban expansion by property development, as well as by the subsidization of manufacturing firms in industrial parks, while financing infrastructure extending to the urban fringes [14].

(2) Land finance drives urban land expansion. To obtain more revenue and accelerate economic growth, local governments often supply land to the market in seemingly contradictory ways. Industrial land is usually sold to enterprises at the lowest possible prices. In regions with a stronger dependence on land finance, local governments tend to sell commercial and residential land in highly marketable ways to rapidly compensate for fiscal deficits [39]. The supply of industrial land is conducive to industrialization, thus helping cities to attract more immigrants, develop tertiary industries, and increase the demand for residential and commercial land. Furthermore, urban sprawl in China is manifested in multiple forms, such as leapfrogged industrial parks [21], which, with other industrial land, are distributed in the outskirts of cities, thus directly reflecting the characteristics of urban land expansion.
(3) Economic growth drives urban land expansion by generating demand. Economists believe that population growth, rising household incomes, and transportation improvements are responsible for this spatial growth [42], which can explain urban sprawl in the United States and developing countries, such as China [43]. Economic growth has affected all three variables. First, economic growth drives the development of nonagricultural industries in urban areas, which attracts many rural people to migrate to cities. Furthermore, economic growth promotes scientific and technological progress, increases agricultural labor productivity, and enables surplus laborers in rural areas to seek employment opportunities in cities. Second, economic growth increases household incomes, which foster urban growth because urban residents demand more living spaces as they become more affluent [1]. Finally, only economic growth can help cities to afford better, cheaper, and more convenient public transportation, which leads to the settling of more residents in the outskirts of cities as the optimal choice between transportation and housing costs, thereby stimulating the expansion of the cities.
(4) Urban land expansion promotes economic growth through the contribution of input factors. In neoclassical theories of endogenous economic growth, the roles of scientific and technological progress are valued; however, land is usually not included because of its fixed supply and substitution by other factors. China’s urban economy differs from the developed economy because local governments can sell and lease land-use rights, and then retain revenues. Moreover, they have the right to expropriate rural collective land. The effects of urban land expansion on economic growth through the contribution of inputs are as follows: First, the expansion of urban land leads to the increase in the available spaces for residents and industries, thus concentrating labor, capital, and other factors in urban areas, which benefits the development of industry and promotes economic growth. Second, the expansion of urban land changes economics of scale and agglomeration, which affects land prices and the costs of infrastructure construction, transportation, and public services, thus influencing economic growth [44].
(5) Land finance influences economic growth through investment. In addition to private investment, government investment is also an important driving force. Under financial marketization, the government must find ways to raise large amounts of funds. When obtaining loans from banks, governments should possess assets with strong value preservation and appreciation for collateral value. Land and land revenues owned by local governments are excellent collateral. The continuous growth of land finance and the resultant increase in local financial revenue have contributed to infrastructure construction and the supply of public services, through which local governments have gained significant momentum in accelerating urbanization, attracting more investments, and promoting local economic development [45,46].
(6) Economic growth facilitates the development of the real-estate market in order to increase the scale of land finance. Rapid economic growth has improved the people’s living standards, so that they can afford larger houses, higher-quality commodities, and more luxury goods, which stimulates the demand of enterprises for land. In the case of a relatively short supply of urban land, local governments sell land to obtain as much revenue as possible. Second, hikes in land prices driven by economic growth also increase the prices of buildings, which in turn increase tax revenues on land and building transactions, thereby also increasing land finance.

## 3. Model Settings and Data Description

### 3.1. Model Settings

The above-mentioned theoretical framework indicates that the relationships among urban land expansion, land finance, and economic growth are complex and indicate endogenous causality. Therefore, herein, a PVAR model was constructed to avoid this endogenous problem. PVAR is a type of model that is highly relevant to the concurrent condition model. Furthermore, it can effectively control estimation bias caused by spatial and individual heterogeneity compared with other traditional econometric technologies [47]. The general expression of the PVAR model is:

(1) $$\;Y_{it}=\gamma_{0}+\overset{p}{\underset{j=1}{\sum }}\gamma_{j}Y_{i,t-j}+\alpha_{i}+\beta_{t}+\varepsilon_{it}$$

where *i* and *t* refer to province and time, respectively; $Y_{it}$ indicates the endogenous variables, which change with time and region; $\gamma_{0}$ and $\gamma_{i}$ are the estimated coefficients of the constant term and the lagged endogenous variable, respectively; p is the lag period; $\alpha_{i}$ and $\beta_{t}$ are the vectors of the individual and time effects, respectively; and $\varepsilon_{it}$ is the random disturbance term. Urban land expansion, land finance, and economic growth were represented in this study by the respective logarithmic quantities, namely lnland, lnfin, and lngdp.

The following five steps are generally involved in the construction of the PVAR model:

First, the panel unit-root test is applied to investigate the stationarity of the related variables in order to ensure the suitability of the PVAR model. The equations for setting the panel-stability test are as follows:

(2) $$y_{it}=\rho_{i}y_{it-1}+\Delta_{i}x_{it}+\varepsilon_{it}$$

where *i* = 1, 2, …, *N* represent provinces observed over periods *t* = 1, 2, …, *T*; $x_{it}$ are exogenous variables including any fixed effects; and $\rho_{i}$ denotes the autoregressive coefficients. If $\rho_{i}<1$, $y_{i}$ is said to be weakly trend-stationary. Conversely, if $\rho_{i}=1$, $y_{i}$ has a unit root. $\varepsilon_{it}$ represents the stationary error terms.

In this study, five different tests were employed. These were described by Levin–Lin–Chu (LLC) (2002), Breitung (2000), Im–Pesaran–Shin (IPS) (2003), a modified version of the test described by Dickey and Fuller (ADF) (1979), and a test described by Phillips and Perron (PP) (1988). The null hypothesis of LLC is that the data have a common unit-root process. IPS, ADF, and PP assume that the data have an individual unit-root process.

Secondly, the optimal lag period of PVAR model was determined by using the Akaike’s Information Criterion (AIC), the Bayesian Information Criterion (BIC), and the Hannan and Quinn Information Criterion (HQIC). Based on the above-stated conclusion, the System-GMM method was applied to estimate PVAR model.

Thirdly, the equations for the Granger causality test are as follows:

(3) $$y_{it}=\gamma +\sum_{m=1}^{p}\alpha_{m}y_{i,t-m}+\sum_{m=1}^{p}\beta_{m}x_{i,t-m}+u_{i}+\varepsilon_{it}$$

where $y_{it}$ denotes the dependent variable, *p* is the optimum lag length determined by the AIC criterion, $u_{i}$ is the individual effect, $\varepsilon_{it}$ is the random disturbance, and $\alpha_{m}$ and $\beta_{m}$ are the regression coefficients. Obviously, it is a dynamic-panel model and should be estimated by using differential GMM or system GMM. The null hypothesis H0: $\beta_{1}=\beta_{2}=\ldots =\beta_{p}=0$ is rejected; thus, it could be concluded that there is a Granger causality from *x* to *y*.

Fourth, once all the coefficients of the PVAR model are estimated, the Cholesky decomposition is used to compute the impulse-response functions (IRFs).

Finally, in this study, variance decompositions were also presented, which show the percentage of the variation in one variable that is explained by the shock to another variable. These variance decompositions accumulate over time and show the magnitude of the total effect. The total effect accumulated over the 1, 5, 10, 15, and 20 years is presented in this study. Herein, the Stata 15.1 was used to analyze the data.

### 3.2. Data and Variables

The panel data from 2000 to 2017 for 30 provinces in mainland China were used herein (Table 1). Tibet, Hong Kong, Macao, and Taiwan were excluded because of data unavailability. The original data of urban construction land areas were collected from the China Statistical Yearbook (2009–2018) and the China City Statistical Yearbook (2001–2008). The missing individual annual data were calculated by using the average value of the year before and the year after. When this value was lacking for two or more consecutive years, the weighted average method was used.

Land finance includes land-transfer revenue and land-related tax revenue. Data on land-transfer revenue were obtained from the China Land and Resources Statistics Yearbook (2010–2018) and China Land and Resources Yearbook (2001–2009). Land-related taxes include land value-added, urban land-use, farmland occupation, property, and deed taxes. These data were obtained from the Finance Yearbook of China (2001–2018). To eliminate the influence of price factors, land finance was deflated by the consumer price index (CPI), the values of which were collected from the China Statistical Yearbook (2001–2018).

The level of economic growth was represented by the GDP deflated by the GDP index with the base year 2000. The data for nominal GDP are available in the China Statistical Yearbook (2001–2018) and the GDP index was obtained from the statistical yearbook of each province.

**Figure 4:**
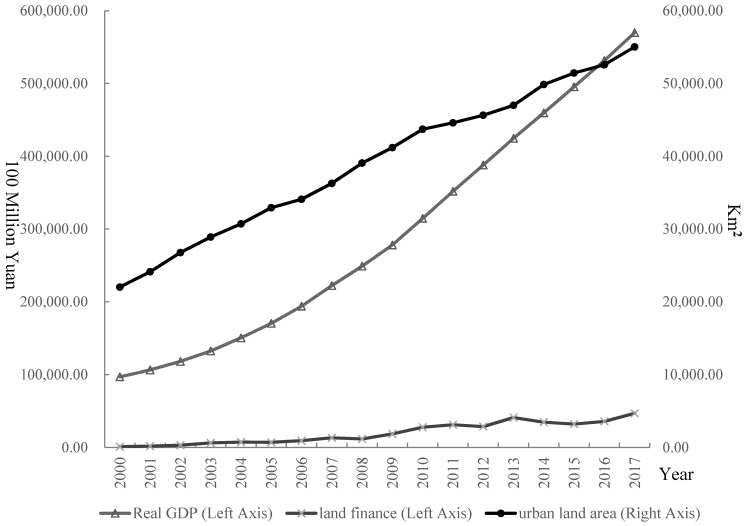
Real GDP, land finance, and urban land construction area from 2000–2017 in China.

Figure 4 presents the characteristics of the temporal change of urban land expansion, land finance, and economic growth from 2000 to 2017. GDP increased from CNY 9709.19 billion in 2000 to CNY 56,995.5 billion in 2017, which represented an average annual growth rate of 10.97%. Similarly, urban construction land and land finance increased by 5.54% and 25.09%, respectively. Therefore, the changes in urban land expansion, land finance, and economic growth exhibited similar trends.

## 4. Empirical Results and Discussion

### 4.1. Panel Unit-Root Test

To avoid the possible shortcomings and defects of a single testing method, this study applied the LLC test, the Breitung test, the IPS test, the Fisher-ADF test, and the Fisher-PP test. In general, when the raw sequence was nonstationary, the first-order difference sequence was used to make it stationary. When a given data sequence passed all the above-mentioned tests, then it was stationary; otherwise, it was nonstationary. The results of the panel unit-root test are presented in Table 2. Most of the values of lnland, lnfin, and lngdp were stationary, thus contradicting the null hypothesis of the existence of a unit root at a 1% level of significance. Therefore, it was concluded that all the raw data series could be applied to the empirical analysis.

### 4.2. PVAR Model Regression Analysis

Before the implementation of PVAR model, its optimal lag period must be determined. Based on the study by Andrews and Lu [48], in this study, comprehensive use of the AIC, the BIC, and the HQIC was made. Table 3 summarizes that when the lag period is 1, the absolute values of BIC and HQIC reach their minimum values. In contrast, the absolute value of AIC reaches the minimum when the lag period is 2. When the results obtained by the three criteria are inconsistent, the BIC criteria and the HQIC criteria should yield better results compared to the AIC criteria. Hence, 1 is the lag period selected to establish the PVAR (1) model in this study.

Table 4 presents the estimated results based on System-GMM. When lnland is used as the explanatory variable, a significant correlation exists between the variables and its first-period lag, indicating that the lnland in China is dependent on its own inertia. Similarly, China’s land finance and economic growth also exhibit their own development inertia. The first-period lags of both lnfin and lngdp exhibit positive, but insignificant, effects on lnland. The first-period lag of lnland shows negative, but insignificant, effects on lnfin. However, for every 1% increase in lngdp, lnfin increases by 0.0399%. Therefore, lngdp is negatively affected by lnland but positively affected by lnfin. In summary, it was concluded that urban land expansion, land finance, and economic growth affect each other, which is a conclusion consistent with our theoretical analysis.

### 4.3. Granger Causality Test

Table 5 reports the details of the Granger causality test. When Δlnland is used as the dependent variable, the *p*-value is found to be significant (at the 1% level) only in the case of Δlnfin, indicating that land finance is one of the Granger causes of urban land expansion. Our result is quite different from that reported by Sudhira et al. (2004), who found that urban sprawl was a global phenomenon driven mainly by population growth and large-scale migration [49]. Seto et al. (2010) [7] argued that urban land expansion in China was mostly driven by urban population growth. A causality relationship from GDP to urban land expansion was nonexistent. When Δlnfin was used as the explained variable, the statistic was never significant. Specifically, urban land expansion cannot provide a full explanation for how land finance arises. There are at least two reasons for this. First, the land supplied to the market includes plots in built-up and newly expanded urban areas. The former has prime locations and differential rents resulting in high prices, whereas the latter are farther away from the central business center, resulting in relatively low prices. Second, the plots in the newly expanded urban areas constitute a small proportion of the entire urban area. Economic growth is not the direct Granger cause of land finance. In contrast, urban land expansion and land finance are both the Granger causes of economic growth at the 1% significance level. The results show that urban land is a very important factor of production in China, as it not only provides space for production, but also accumulates funds for cities, enhances the investment capacities of urban governments, and affects China’s economic growth. The results also lay a theoretical foundation for how urban governments could manage economic development by the utilization of land resources.

In summary, the Granger causality test has helped to solve the three research issues. First, the empirical results confirm that urban land expansion is driven mainly by the local government’s pursuit of land finance. Second, land finance is highly correlated to economic growth but not to the results of urban land expansion. Third, land is a very important input factor for the urban economies of China. Urban land expansion and land finance both affect economic growth.

### 4.4. Impulse-Response Analysis

**Figure 5:**
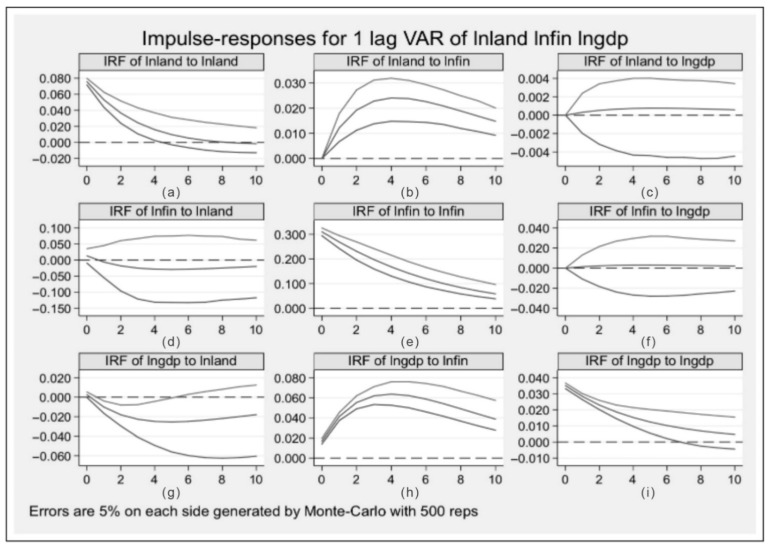
Results of impulse responses.

The main objective of impulse-response analysis is to visualize the dynamic impulse process among urban land expansion, land finance, and economic growth in China. Figure 5a shows that the response degree of lnland is usually greater than 0, indicating that China’s urban land expansion continuously and positively affects itself. However, the cumulative effects decrease in successive years. Figure 5b shows the response of lnland to a standard deviation in lnfin. The response first increases, then decreases. Figure 5c shows that after receiving a standard-deviation impulse from lngdp, lnland rarely responds during all 10 periods. For a standard-deviation impulse of lnland, lnfin responds positively in the first period before turning negative and remaining stable (Figure 5d). Figure 5e demonstrates that faced with a standard-deviation impulse from lnfin, the response degree of lnfin decreases from 0.3001 to 0.0054. During all the 10 periods, the response degree of lnfin to lngdp remains insignificant (Figure 5f). Figure 5g shows that lnland offers a negative response to lngdp, first increasing and then stabilizing. Figure 5h shows that lngdp exhibits a definitive positive response to lnfin and presents an “inverted U-shaped” curve. Figure 5i shows the impulse effect of lngdp on itself. Exposed to a standard-deviation impulse from itself, the response degree of lngdp is greater than 0, which indicates that China’s economic development continuously and positively affects itself, but the effect has been decreasing gradually.

### 4.5. Variance Decomposition

The variance-decomposition approach was employed to analyze the contributions of various structural impulses to the fluctuations in urban land expansion, land finance, and economic growth. Table 6 presents that from the 1st period to the 20th period, the explanatory power of lnland for itself decreases from 100% to 70.50%. Moreover, the explanatory power of lnland to lnfin increases from 0.20% to 1.90%. In contrast, changes in lngdp make the lowest contributions to changes in lnland and lnfin. In terms of the explanatory power of each impulse variable on lngdp, lnfin shows the highest contribution rate to the changes in lngdp, whose average contribution rate is 65.26%. The average contribution rates of lnland and lngdp are 9.52% and 25.24%, respectively. In the first period, the explanatory powers of lnland, lnfin, and lngdp on the changes in lngdp are 0.40%, 18.50%, and 81.10%, respectively. With the passage of time, both the explanatory powers of lnland and lnfin on the changes in lngdp increase. However, the inertia of economic development on its own dependence gradually decreases.

## 5. Conclusions and Policy Implications

The primary objective of this study was to systematically reveal the relationships among urban land expansion, land finance, and economic growth in China. Herein, panel data from 2000 to 2017 for 30 provinces in mainland China were used to study the relationships by employing a PVAR model. The empirical results confirmed that the factors affected each other.

A Granger causality Test revealed that lnland and lnfin could explain the changes in lngdp to some extent, but not vice versa. Land finance was one of the Granger causes of urban land expansion at the 1% significance level. Furthermore, the interactions among urban land expansion, land finance, and economic growth in China constitute a dynamic impulse process. The variance decomposition showed downward trends in the explanatory powers of lnland, lnfin, and lngdp for themselves. Moreover, the explanatory power of lnland for lnfin increased from 0.20% to 1.90%. In contrast, changes in lngdp made the lowest contributions to changes in lnland and lnfin. In terms of the explanatory power of each impulse variable for lngdp, lnfin made the highest contribution to changes in lngdp, whose average contribution rate was 65.26%.

Our findings have important policy implications.

(1) Urban land expansion and land finance both affect economic growth. A rational view of the negative externalities accompanied by such expansion should be considered. First, urban land expansion is an inevitable phenomenon resulting from economic development and urbanization. Urban land has a higher efficiency than rural land; therefore, control of this expansion is critical. Regulatory policies should focus on preventing over rapid or excessive expansion and urban sprawl. Second, the dual land-ownership system has made urban expansion achievable through the expropriation of rural collective land. Government’s power to expropriate should be strictly limited to serving the public interest, with farmers and collectives receiving market prices as compensation. The government should not treat all land development as public interest. Third, governments should improve the efficiency of construction land, in particular, industrial land, which should be allocated by market-oriented means, and establish a mechanism for the withdrawals and transfers of rights to industrial land. Finally, public policies’ control of urban size according to the predicted urban population is beneficial because it delineates and implements a moderately flexible urban-growth boundary, as well as establishes a transaction mechanism for new construction-land indicators among the urban governments.
(2) The pursuit of land finance by local governments has driven the expansion of urban land. Therefore, we should dialectically examine the problems faced by local governments that are highly dependent on land finance. With the development of the economy and the reform of the local tax system, the phenomenon of land finance may disappear. However, attention should be paid to the fact that overreliance on land finance in cities leads to the boom of the real-estate industry and spurred market bubbles that drive prices beyond their fair values. Therefore, herein, two suggestions are offered. First, the responsibilities of local governments, in particular, for economic development, should be reduced. Moreover, transfer payments from central governments to local governments should be increased to eliminate the fiscal gap and reduce the incentives for local governments to pursue land finance. Second, taxes and fees should be reformed to increase local government revenues. Lessons can be learnt from the experiences of the United States and other developed countries where property taxes are imposed. Property tax accounts for about more than half of the revenues raised by the local governments. At present, feasible measures include reforming the Chinese property tax system, broadening its base, and raising the ceiling rates.

To contribute to the field of land economics, our novel approach incorporated urban land expansion, land finance, and economic growth into one system to analyze the relationships among them. Herein, it was found that there exists multidirectional Granger causality among these three variables, which is more in line with China’s complex economic environment. Urban land expansion and land finance are driving factors of economic growth, which can explain why the local governments are so keen to intervene the land market.

Admittedly, this study has several limitations. For example, first, economic growth is affected by several factors. More variables should be incorporated into the system composed of land finance, urban expansion, and economic growth. Second, given that the three variables analyzed represent a complex system, whose working ought to be analyzed via much more intricate model structures. Future research can improve the accuracy of the demonstration by using structural equation models and system simulation technology. Finally, the revenues obtained by local governments using land as collateral have not been considered in the measurement of land finance because of limitations in accessing the relevant data. Although these loan repayments have been included in projections of future fiscal revenues, they can increase the budgets of current local governments and investments in infrastructure construction and urban environments, as well as contribute to economic development.

## Figures and Tables

**Table 1:** Variable measurement and data source.

Variable	Measurement	Data Source
Urban land expansion	Urban construction land area	China Statistical Yearbook and China City Statistical Yearbook
Land finance	Land-transfer revenue and land-related tax revenue	China Land and Resources Statistics Yearbook, China Land and Resources Yearbook, and Finance Yearbook of China
Economic growth	Real GDP	China Statistical Yearbook and statistical yearbook of each province

**Table 2:** Panel unit-root tests.

Series	Test Type				
	LLC Test	Breitung Test	IPS Test	Fisher-ADF	Fisher-PP
lnland	−7.3762 *** (0.0000)	0.8928 (0.8140)	−1.8780 ** (0.0302)	164.6123 *** (0.0000)	193.3150 *** (0.0000)
lnfin	−4.9013 *** (0.0000)	−0.3642 (0.3578)	−4.3316 *** (0.000)	161.6238 *** (0.0000)	155.8744 *** (0.0000)
lngdp	−6.1138 *** (0.0000)	1.1232 (0.8693)	−3.3321 *** (0.0004)	174.5040 *** (0.0000)	19.1564 *** (1.0000)

Notes: All panel unit-root tests show restricted intercepts and trends for all variables. **, and *** represent the levels of significance at 5%, and 1%, respectively. The *p*-values are in brackets.

**Table 3:** Results of multicriteria test.

Lag	Test Criteria			Conclusion
	AIC	BIC	HQIC	
1	−5.2781	−4.4173 *	−4.9397 *	Lag 1
2	−5.2815 *	−4.2953	−4.8928	
3	−4.4025	−3.2770	−3.9576	

Notes: * represents the level of significance at 10%.

**Table 4:** Regression results of PVAR model.

		Coef.	Std. Err.	Z	p > |Z|	95% Conf. Interval
lnland	L1.lnland	0.6954	0.0777	8.9500	0.0000	(0.5431, 0.8477)
	L1.lnfin	0.0387	0.0120	3.2300	0.0010	(0.0152, 0.0622)
	L1.lngdp	0.0090	0.0382	0.2300	0.8150	(−0.0659, 0.0838)
lnfin	L1.lnland	−0.2334	0.3497	−0.6700	0.5040	(−0.9189, 0.4519)
	L1.lnfin	0.8641	0.0488	17.7100	0.0000	(0.7685, 0.9597)
	L1.lngdp	0.0399	0.1983	0.2000	0.8410	(−0.3488, 0.4285)
lngdp	L1.lnland	−0.1761	0.0391	−4.5000	0.0000	(−0.2527, −0.0995)
	L1.lnfin	0.0894	0.0055	16.2600	0.0000	(0.0786, 0.1001)
	L1.lngdp	0.8078	0.0210	38.4500	0.0000	(0.7666, 0.8490)

Note: L1 represents the variable of the first-period lag.

**Table 5:** Panel Granger causality tests.

Dependent Variable	Explanatory Variable	Chi2	O-Value
Δlnland	Δlnfin	10.4340	0.0000
	Δlngdp	0.0550	0.8150
	All	12.7670	0.0020
Δlnfin	Δlnland	0.4458	0.5040
	Δlngdp	0.0405	0.8410
	All	1.0907	0.5800
Δlngdp	Δlnland	20.2900	0.0000
	Δlnfin	264.5000	0.0000
	All	291.1700	0.0000

**Table 6:** Variance decomposition.

Response Variable	Period (Years)	Impulse Variable		
		lnland	lnfin	lngdp
lnland	1	1.0000	0.0000	0.0000
	5	0.8690	0.1310	0.0000
	10	0.7430	0.2570	0.0000
	15	0.7110	0.2890	0.0000
	20	0.7050	0.2950	0.0000
lnfin	1	0.0020	0.9980	0.0000
	5	0.0070	0.9930	0.0000
	10	0.0150	0.9840	0.0000
	15	0.0180	0.9810	0.0000
	20	0.0190	0.9810	0.0000
lngdp	1	0.0040	0.1850	0.8110
	5	0.0890	0.7330	0.1780
	10	0.1210	0.7790	0.1000
	15	0.1300	0.7830	0.0880
	20	0.1320	0.7830	0.0850

## Data Availability

Not applicable.
